# Cost-Effectiveness Analysis on Endoscopic Surveillance Among Western Patients With Barrett's Esophagus for Esophageal Adenocarcinoma Screening: Erratum

**DOI:** 10.1097/01.md.0000489121.76566.78

**Published:** 2016-07-08

In the article “Cost-Effectiveness Analysis on Endoscopic Surveillance Among Western Patients With Barrett's Esophagus for Esophageal Adenocarcinoma Screening”,^1^ which appeared in Volume 94, Issue 39 of *Medicine*, an incorrect doi was linked to the article. The article has since been corrected online.
